# Impact of phase angle and sarcopenia estimated by bioimpedance analysis on clinical prognosis in patients undergoing hemodialysis: A retrospective study

**DOI:** 10.1097/MD.0000000000029375

**Published:** 2022-06-24

**Authors:** Eunjin Bae, Tae Won Lee, Wooram Bae, Seongmin Kim, Jungyoon Choi, Ha Nee Jang, Se-Ho Chang, Dong Jun Park

**Affiliations:** a Department of Internal Medicine, Gyeongsang National University Changwon Hospital, Changwon, South Korea; b Department of Internal Medicine, Gyeongsang National University College of Medicine, Jinju, South Korea; c Institute of Health Science, Gyeongsang National University, Jinju, South Korea; d Department of Internal Medicine, Gyeongsang National University Hospital, Jinju, South Korea.

**Keywords:** bioimpedance analysis, hemodialysis, muscles, nutrition assessment, prognosis

## Abstract

Bioimpedance analysis (BIA) has been widely used in the evaluation of body composition in patients undergoing maintenance hemodialysis. We conducted this study to evaluate impact of phase angle (PA) and sarcopenia measured by BIA on clinical prognosis in these patients.

This longitudinal retrospective study enrolled patients who underwent hemodialysis between January 2016 and March 2019. The patients were stratified into higher (> 4°) and lower (≤ 4.0°) PA groups. Sarcopenia was defined when the appendicular skeletal muscle mass was < 20 kg in men and < 15 kg in women.

Of the 191 patients, 63.4% were men. The mean age was 64.2 ± 12.4 years. The lower PA group was older, had a higher proportion of women, a lower body mass index, lower albumin, cholesterol, uric acid, and phosphorus levels, and a higher incidence of history of coronary artery disease than the higher PA group. Linear regression analysis revealed that PA was significantly associated with body mass index (*B* = 0.18, *P* = .005), serum albumin (*B* = 0.23, *P* = .001), and creatinine levels (*B* = 0.32, *P* < .001). During a median follow-up of 16.7 months, 14.1% (n = 27) of patients experienced major adverse cardiovascular events and 11.0% (n = 21) died. Kaplan–Meier survival analysis showed that the higher PA group had significantly better survival, regardless of sarcopenia. Multivariate Cox analyses revealed that lower PA (0.51 [0.31–0.85], *P* = .010), higher IDWG (1.06 [1.01–1.12], *P* = .028) and C-reactive protein level (1.01 [1.01–1.02], *P* < .001), and a history of coronary artery disease (3.02 [1.04–8.77], *P* = .042) were significantly related to all-cause mortality after adjusting for other covariates.

PA measured by BIA was an independent factor in the prediction of mortality in maintenance hemodialysis patients, regardless of sarcopenia. Intervention studies are needed to confirm if the improvement in PA is associated with better clinical outcome.

## 1 Introduction

It is known that survival of incident dialysis patients is even lower than that of patients with various types of solid-organ cancers, such as prostate and colorectal cancer in men and breast and colorectal cancer in women.^[1]^ Malnutrition usually affects 30% to 70% of maintenance hemodialysis (MHD) patients, depending on the nutritional assessment method used, and is a significant factor in quality of life and survival.^[2,3]^ The nutritional status of MHD patients can be assessed based on clinical symptoms, questionnaire data, the subjective global assessment, body mass index (BMI), laboratory data (e.g., serum albumin, prealbumin, cholesterol, and creatinine levels), dual-energy X-ray absorption spectrometry (DEXA), and body composition methods such as bioimpedance analysis (BIA).^[4–7]^ Phase angle (PA) measured by BIA reflects the nutritional status of patients with chronic kidney disease ^[8–10]^ and has prognostic value for renal patients.^[11–13]^

Sarcopenia is currently defined as a generalized loss of skeletal muscle mass combined with reduced strength or physical performance.^[14–16]^ Sarcopenia pathophysiology in CKD is complex and might be associated with uremic toxins, oxidative stress, insulin resistance, malnutrition, and physical inactivity.^[17–19]^ In patients undergoing dialysis, sarcopenia has a prevalence of between 20% to 55% and is associated with an increased mortality risk.^[20–22]^ Dialysis itself might increase the prevalence of sarcopenia in MHD patients due to the accelerated protein catabolism induced by metabolic acidosis, unresolved uremia, and elevated pro-inflammatory cytokine levels.^[23]^ Previous studies have shown that BIA provides reliable skeletal muscle mass measurements in end-stage renal disease patients.^[24,25]^

There have been many studies in which PA and sarcopenia via BIA affect clinical prognosis in patients undergoing dialysis individually, but there has been no study on how combination of these two factors affects clinical prognosis. Therefore, we conducted this study to evaluate impact of PA and sarcopenia by BIA on clinical prognosis in patients undergoing maintenance hemodialysis.

## 2 Materials and methods

### 2.1 Study population and data collection

This longitudinal retrospective study initially involved 314 MHD patients starting hemodialysis (HD) between January 2016 and March 2019 at Gyeongsang National University Changwon Hospital, South Korea. Outpatients who underwent MHD for at least 12 weeks, and were ≥ 18 years of age, were included. All patients received HD three times per week via vascular access, and each dialysis session was at least 3 hours 30 min in duration. Active cancer patients and those with amputations or body deformities were excluded; 30 patients who died within 8 weeks, and 93 without BIA data, were excluded. The institutional review board (IRB) of Gyeongsang National University Changwon Hospital approved research protocol for a retrospective analysis of the collected data (IRB #201812012). IRB approved the exemption from obtaining written informed consent because the research was a medical record-based retrospective analysis and the included patients were anonymized.

In total, 191 patients undergoing MHD treatment at our HD center or regional HD clinics were included in the study. Baseline measurements (including laboratory data) were taken at enrollment. The patients were followed up regarding survival time since enrolling in the study, and disease status was determined based on a detailed medical history. A single clinician reviewed each patient's medical records and extracted all relevant clinical data, including age, sex, height, weight, systolic blood pressure, diastolic blood pressure, comorbidities, BMI, and BIA data. We obtained the mortality data from Statistics Korea. BMI was calculated as the post-dialysis weight (kg)/height (m^2^). Blood samples for hemoglobin, total protein, albumin, creatinine, uric acid, C-reactive protein, calcium, phosphorus, intact PTH, and ferritin were done just before hemodialysis start.

### 2.2 Phase angle and muscle mass measurements

BIA-derived PA was measured using the InBody S10 (Biospace, Seoul, Korea) body composition analyzer, which is a multifrequency bioimpedance device. BIA was performed on HD patients by the same operator 30 min after the dialysis sessions. All patients were placed in the supine position with the legs set apart and arms not in contact with the torso. Two pairs of electrodes were used: the first pair was placed on the dorsum of the hand over the third metacarpophalangeal joint and the wrist, and the second pair was placed on the ipsilateral third metatarsophalangeal and ankle joints. The whole-body PA was measured at a frequency of 50 kHz, which is the most commonly used frequency. The PA was calculated automatically by the BIA device based on two components, resistance (R) and reactance (Xc), using the following formula: PhA (°) = arctangent (Xc/R) × (180/π). The cut-off value of PA chosen to evaluate mortality in our study was 4.0. This value has been used in a previous study.^[26]^

### 2.3 Clinical outcomes and sarcopenia diagnosis

The primary outcome was all-cause mortality according to the PA and sarcopenia during follow-up. Major adverse cardiovascular events (MACE) was also evaluated, as well as nonfatal stroke, nonfatal myocardial infarction, and cardiovascular death.^[27]^ BIA yields objective indicators of muscle mass. Appendicular skeletal muscle mass (ASMM) was calculated as the sum of the skeletal muscle in the arms and legs, and was determined automatically by the BIA device. Sarcopenia was considered present when the ASMM was <20 kg in men and <15 kg in women.^[15]^

### 2.4 Statistical analysis

All continuous variables are expressed as means and standard deviations, while categorical variables are expressed as percentages. Patients were stratified into higher (> 4°) and lower (≤ 4.0°) PA groups. The PA groups were compared using the chi-square test for categorical variables and the analysis of variance and *t*-test for continuous variables. Linear regression analyses were performed to evaluate the relationships of clinical and biochemical parameters with PA. Survival curves were plotted using the Kaplan–Meier survival method and *P*-values were calculated using the log-rank method. Cox regression analyses were performed to identify risk factors for all-cause mortality, and to investigate the risk of MACE. Logistic regression analysis was used to identify risk factors for sarcopenia. Statistically significant (*P* < .10) and clinically important variables were entered into the multivariate stepwise Cox regression, linear regression, and logistic regression models. Variables were selected using the backward conditional method. SPSS for Windows software (ver. 25.0; IBM Corp., Armonk, NY) was used to perform the statistical analysis. *P*-values < .05 were considered significant.

## 3 Results

### 3.1 Baseline characteristics according to phase angle

**Figure 1:**
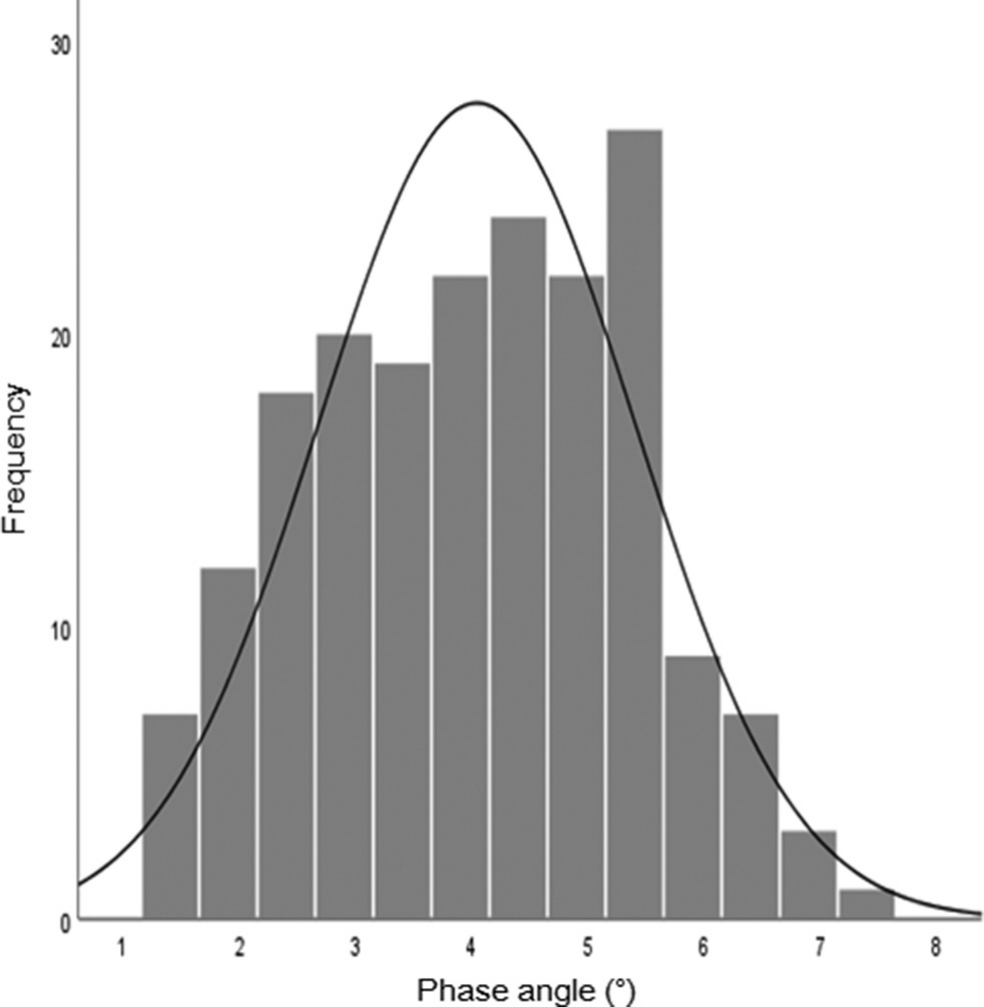
Distribution of the phase angle in maintenance hemodialysis patients.

In total, 191 patients undergoing MHD treatment at our HD center or regional HD clinics were included in the study. The initial clinical and laboratory values of the patients are shown in Table 1. The mean age was 64.2 ± 12.4 years and 63.4% of the patients were men. The average number of months on HD at enrollment was 14.8. The mean PA was 4.0 ± 1.4°, and 60 patients had sarcopenia (31.4%). Figure 1 and Table 1 show the PA data of the patients. The lower PA group was significantly older, had a higher proportion of women, a higher incidence of history of coronary artery disease (CAD), higher interdialytic weight gain (IDWG), sarcopenia, higher C-reactive protein (CRP), ferritin levels, and lower ASMM. Nutrition-related factors, such as the BMI and the albumin, cholesterol, uric acid, and phosphorus levels, were significantly higher in the higher PA group than in the lower PA group (Table 1).

**Table 1:** Baseline characteristics according to phase angle.

Variables	Total (N = 191)	Phase angle≤ 4.0 (N = 97)	Phase angle > 4.0 (N = 94)	*P* value
Age (yr)	64.2 ± 12.4	68.5 ± 10.5	59.8 ± 12.7	<.001
Men (N, %)	121 (63.4)	51 (52.6)	70 (74.5)	.002
BMI (kg/m^2^)	22.0 ± 3.1	21.3 ± 3.3	22.7 ± 2.8	.002
ASMM (kg)	18.1 ± 6.6	16.3 ± 7.5	20.0 ± 4.8	<.001
Phase angle (°)	4.0 ± 1.4	2.9 ± 0.8	5.2 ± 0.7	<.001
Dialysis vintage (month)	14.8 (0.3–373.0)	12.7 (0.59–142.9)	18.2 (0.33–373.0)	.856
IDWG (% of body weight)	6.6 (0.1–89.8)	7.2 (0.3–89.8)	5.4 (0.1–78.8)	.031
SBP (mmHg)	142.5 ± 21.4	141.6 ± 22.2	143.4 ± 20.6	.575
DBP (mmHg)	76.1 ± 12.9	74.5 ± 13.2	77.7 ± 12.4	.087
Hemoglobin (g/dL)	10.1 ± 1.4	10.1 ± 1.3	10.2 ± 1.5	.679
Albumin (g/dL)	3.5 ± 0.7	3.2 ± 0.7	3.7 ± 0.5	<.001
Cholesterol (mg/dL)	146.5 ± 44.1	136.9 ± 45.1	156.0 ± 41.2	.003
Uric acid (mg/dL)	6.6 ± 2.4	6.1 ± 2.3	7.2 ± 2.4	.001
Intact PTH (pg/mL)	156.3 (6.5–644.4)	182.0 (13.1–644.4)	118.2 (6.5–510.1)	.003
Calcium (mg/dL)	8.6 ± 1.1	8.7 ± 1.1	8.6 ± 1.0	.572
Phosphorus (mg/dL)	4.7 ± 1.9	4.2 ± 1.6	5.3 ± 2.0	<.001
CRP (mg/L)	22.8 ± 49.9	34.3 ± 59.7	11.2 ± 33.8	.002
Ferritin	239.8 (15.6–1604.2)	461.7 (18.6–1604.2)	185.9 (15.6–957.8)	<.001
Diabetes mellitus (N, %)	120 (62.8)	63 (64.9)	57 (60.6)	.538
Hypertension (N, %)	177 (92.7)	88 (90.7)	89 (94.7)	.294
CAD (N, %)	88 (46.1)	45 (46.4)	24 (25.5)	.003
CVD (N, %)	30 (15.7)	17 (17.5)	13 (13.8)	.483
Sarcopenia (N, %)	60 (31.4)	45 (46.4)	15 (16.0)	<.001

ASMM = appendicular skeletal muscle mass, BMI = body mass index, CAD = coronary artery disease, CRP = C-reactive protein, CVD = cerebrovascular disease, DBP = diastolic blood pressure, PTH = parathyroid hormone, SBP = systolic blood pressure.

### 3.2 Clinical parameters associated with phase angle

In the MHD patients, PA was positively correlated with nutrition-related factors, such as the BMI (regression coefficient [B] = 0.18, *P* = .005), albumin level (B = 0.23, *P* = .001), and creatinine level (B = 0.32, *P* < .001). However, the serum protein, cholesterol and uric acid levels, and the skeletal muscle mass index (SMI), were not significantly associated with PA. In addition, PA was negatively correlated with age (B =  − 0.21, *P* = .001) and CAD (B =  − 0.17, *P* = .006) (Table 2).

**Table 2:** Association of phase angle with clinical parameters in hemodialysis patients.

	Unadjusted B^a^	*P*	Adjusted B	*P*
Age (yr)	−0.41	<.001	−0.21	.001
Sex (ref. male)	−0.21	.003		
BMI (kg/m^2^)	0.28	<.001	0.18	.005
ASMM (kg)	0.28	<.001		
IDWG (% of body weight)	−0.19	.015		
Total protein (g/dL)	0.20	.006		
Albumin (g/dL)	0.45	<.001	0.23	.001
Cholesterol (mg/dL)	0.22	.002		
Uric acid (mg/dL)	0.26	<.001		
Phosphorus (mg/dL)	0.28	<.001		
BUN (mg/dL)	0.35	<.001		
Creatinine (mg/dL)	0.52	<.001	0.32	<.001
CRP (mg/L)	−0.31	<.001		
Ferritin (ng/mL)	−0.35	<.001		
Diabetes mellitus (yes)	−0.10	.183		
Hypertension (yes)	0.10	.156		
CAD (yes)	−0.25	.001	−0.17	.006
CVD (yes)	−0.15	.043		
Sarcopenia (ref. No)	−0.30	<.001		

B^a^, regression coefficient with phase angle. Adjusted for age, sex, BMI, interdialytic weight gain, albumin, cholesterol, uric acid, phosphorus, creatinine, CRP, CAD, CVD, sarcopenia. ASMM, appendicular skeletal muscle mass, BMI, body mass index; BUN, blood urea nitrogen; CAD, coronary artery disease; CVD, cerebrovascular disease; CRP, C-reactive protein; IDWG (% of body weight).

### 3.3 Risk factors for sarcopenia

Next, we explored the factors associated with sarcopenia. About 31.4% of the patients were diagnosed with sarcopenia by BIA (n = 60). Older age (odds ratio [OR] 1.06, 95% CI 1.03–1.10, *P* = .003), female gender (OR 46.58, 95% CI 16.75–129.51, *P* < .010), and lower BMI (OR 0.78, 95% CI 0.67–0.91, *P* *=* .002), were significantly associated with sarcopenia (Table 3).

**Table 3:** Odds ratios for sarcopenia risk factors.

	Univariate		Multivariate	
	Odds ratio (95% CI)	*P* value	Odds ratio (95% CI)	*P* value
Age (yr)	1.05 (1.02–1.08)	.001	1.06 (1.02–1.11)	.003
Sex (ref. Men)	27.75 (12.11–63.59)	<.001	46.58 (16.75–129.51)	<.001
BMI (kg/m^2^)	0.87 (0.78–0.97)	.010	0.78 (0.67–0.91)	.002
Phase angle (°)	0.59 (0.46–0.77)	<.001		
Dialysis vintage (month)	1.00 (1.00–1.00)	.857		
IDWG (% of body weight)	1.02 (0.99–1.05)	.255		
Phosphorus (mg/dL)	0.76 (0.62–0.94)	.010		
Creatinine (mg/dL)	0.76 (0.67–0.86)	<.001		
Diabetes mellitus	1.28 (0.67–2.42)	.458		

Adjusted for age, sex, BMI, phase angle. BMI = body mass index, IDWG = Interdialytic weight gain.

### 3.4 Risk factors for all-cause mortality according to PA and sarcopenia

**Figure 2:**
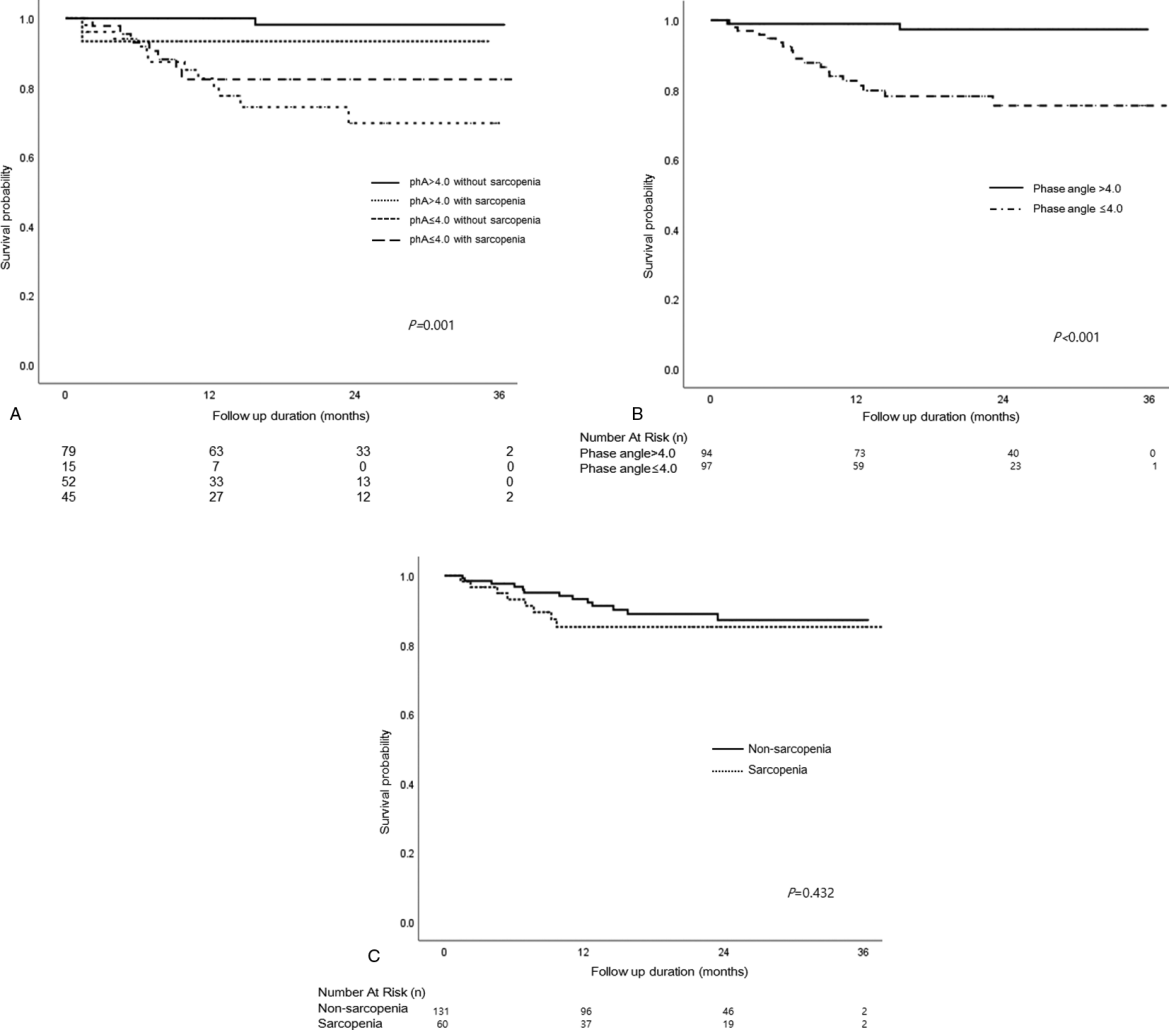
Kaplan-Meier survival probabilities for all-cause mortality based on phase angle (PA) and sarcopenia. (A) High PA without sarcopenia had a lower risk for mortality in patients undergoing hemodialysis. (B) High PA had a significantly lower risk for mortality. (C) Sarcopenia itself did not affect mortality in patients undergoing hemodialysis.

Twenty-one patients (11.0%) died during the 38-month study period, with the higher PA group having a better survival rate than the lower PA group (19.6% vs 2.1%, *P* < .001). Higher PA without sarcopenia had a lower risk for mortality in patients undergoing hemodialysis. Higher PA had a significantly lower risk for mortality irrespective of the absence of sarcopenia. However, sarcopenia itself did not affect mortality in patients undergoing hemodialysis (Fig. 2). We evaluated the risk factors for all-cause mortality using Cox regression analysis. The univariate analysis showed that older age, high IDWG, albumin level, C-reactive protein (CRP) level, and a history of CAD were significantly associated with all-cause mortality. After adjusting for covariates, a lower PA (adjusted hazard ratio [HR] 0.51, 95% confidence interval [CI] 0.31–0.85, *P* = .010), higher CRP level (adjusted HR 1.01, 95% CI 1.01–1.02, *P* < .001), higher IDWG (adjusted HR 1.06, 95% CI 1.01–1.12, *P* = .028), and a history of CAD (adjusted HR 2.78, 95% CI 1.04–8.77, *P* = .042) were significantly associated with all-cause mortality (Table 4).

**Table 4:** Hazard ratios for mortality risk factors.

	Univariate		Multivariate	
	HR (95% CI)	*P* value	HR (95% CI)	*P* value
Age (yr)	1.06 (1.02–1.11)	.004		
Sex (ref. Men)	1.22 (0.51–2.89)	.657		
BMI (kg/m^2^)	0.87 (0.75–1.00)	.048		
Phase angle (°)	0.46 (0.31–0.66)	<.001	0.51 (0.31–0.85)	.010
Dialysis vintage (month)	0.99 (0.96–1.01)	.370		
IDWG (% of body weight)	1.034 (1.017–1.052)	<.001	1.06 (1.01–1.12)	.028
Systolic blood pressure (mmHg)	0.98 (0.96–1.00)	.067		
Albumin (g/dL)	0.37 (0.15–0.92)	.032		
CRP (mg/L)	1.01 (1.01–1.02)	<.001	1.01 (1.01–1.02)	<.001
Diabetes mellitus	0.46 (0.19–1.08)	.074		
CAD	3.87 (1.56–9.59)	.003	3.02 (1.04–8.77)	.042
CVD	1.63 (0.60–4.46)	.338		
Sarcopenia (ref. No)	1.42 (0.59–3.43)	.434		

Adjusted for age, CRP, CAD, phase angle, IDWG, sarcopenia. BMI = body mass index, CAD = coronary artery disease, CRP = C-reactive protein, CVD = cerebrovascular disease, IDWG = Interdialytic weight gain.

### 3.5 Risk factors for major adverse cardiovascular events

Twenty-seven (14.1%) patients experienced MACE during the follow-up period. We performed Cox regression analyses to evaluate the risk factors for MACE. Table 5 shows that lower systolic blood pressure (adjusted HR 0.98, 95% CI 0.96–1.00, *P* = .031) and history of cerebrovascular disease (HR 2.80, 95% CI 1.25–6.28, *P* = .012) were significantly associated with MACE (Table 5).

**Table 5:** Hazard ratios for major adverse cardiovascular events factors.

	Univariate		Multivariate	
	HR (95% CI)	*P* value	HR (95% CI)	*P* value
Age (yr)	1.03 (1.00–1.07)	.067		
Sex (ref. Men)	0.67 (0.29–0.67)	.338		
BMI (kg/m^2^)	0.95 (0.84–1.07)	.389		
Phase angle (°)	0.76 (0.57–1.01)	.061		
Dialysis vintage (month)	1.00 (1.00–1.00)	.301		
IDWG (% of body weight)	1.00 (0.98–1.02)	.992		
Systolic blood pressure (mmHg)	0.98 (0.96–1.00)	.056	0.98 (0.96–1.00)	.031
Albumin (g/dL)	0.89 (0.49–1.64)	.716		
CRP (mg/L)	1.00 (1.00–1.01)	.394		
Diabetes mellitus	1.96 (0.79–4.86)	.149		
CAD	1.77 (0.83–3.77)	.140		
CVD	3.04 (1.35–6.88)	.007	2.80 (1.25–6.28)	.012
Sarcopenia (ref. No)	1.18 (0.53–2.63)	.693		

Adjusted for age, CVD, phase angle, systolic blood pressure, sarcopenia. BMI = body mass index, CAD = coronary artery disease, CVD = cerebrovascular disease, IDWG = Interdialytic weight gain.

## 4 Discussion

Our retrospective study confirmed that the BIA-derived PA, measured at 50 kHz, was associated with biochemical and anthropometric nutritional parameters, such as serum albumin, creatinine, and cholesterol levels, BMI, and clinical parameters such as a history of diabetes mellitus and CAD. The higher PA group had significantly better survival than the lower PA group irrespective of the presence of sarcopenia. A lower PA value, higher CRP level, and history of CAD were significantly associated with all-cause mortality. However, both PA and sarcopenia was not a significant risk factor for MACE.

The importance of BIA in MHD patients is mainly based on the established correlation between PA and mortality.^[2,11,28,29]^ Abad et al showed that the PA, determined by BIA at a frequency of 50 kHz, was a good marker of survival in 164 dialysis patients (127 on HD and 37 on peritoneal dialysis). They reported that patients with a PA > 8° had a significantly higher survival rate at the 6-year follow-up.^[11]^ Segall et al reported a relative risk for mortality of 4.1 in a PA < 6° group compared to a higher PA group.^[28]^ Chertow et al divided more than 3,000 HD patients into PA quintile groups and found that those in the group with the lowest PA had a 1.5 higher relative risk of mortality than those with the highest PA, independent of age, gender, race, diabetes, and serum albumin and creatinine levels.^[2]^ Ruperto et al reported that a PA < 4° was an independent risk factor for mortality in HD patients with protein-energy wasting (PEW).^[26]^ Our data also demonstrated that patients with PA > 4° which is same cut-off value in our study had significantly better survival than those with a PA ≤ 4.0°. The application of different cut-off value of PA in several studies might derive from difference in manufacturing company, ethnicity, and baseline patient's characteristics.

PA measurements by BIA have been widely used to assess nutritional status in patients undergoing chronic hemodialysis because of their simplicity, cost-effectiveness, repeatability, and objective validity.^[8–10]^ However, PA measurements vary among series, probably due to the type of BIA used. One study revealed that BIA-derived 50 kHz PA values were useful to predict PEW in Chinese MHD patients. They showed that BIA-derived PA values were positively associated with nutritional indices, such as biochemical and anthropometric parameters, including albumin, prealbumin, fat-free mass, BMI, and mid-arm muscle circumference. They proposed that PA be used as an important biomarker of PEW in Chinese HD patients; a cutoff value of 4.6° was suggested.^[13]^ Our study used the same type of BIA analyzer and showed that the PA was significantly correlated with several biochemical and anthropometric parameters in Korean MHD patients. BIA-derived PA has potential utility for early detection and monitoring of malnourishment in MHD patients.

The importance of sarcopenia as an independent predictor of mortality in dialysis populations is controversial.^[24,25]^ Ren et al showed that sarcopenia (diagnosed based on SMI muscle mass and strength) and handgrip strength were significantly associated with mortality in 131 MHD patients during a 1-year follow-up. They reported that dialysis duration, diabetes, serum phosphorus level, and malnutrition were independent risk factors for sarcopenia.^[20]^ However, another study of 648 MHD patients reported that neither sarcopenia nor low muscle mass alone were better predictors of mortality than functional limitations, such as slow gait speed and low muscle strength in patients receiving HD during a 1.9-year follow-up.^[25]^ Differences in mortality rates might be due to differences in age, race, dialysis vintage, follow-up period, sarcopenia definition, and diabetes prevalence in enrolled patients. Our study did not show that sarcopenia alone was an independent predictor of mortality in MHD patients. This negative result might be because only muscle mass, and not muscle strength or physical activity measures (such as handgrip strength and gait speed), were assessed in our study.

The characteristics of our study exist in assessing the mortality in patients undergoing MHD through both PA and sarcopenia via BIA whereas other studies used individual factor of PA ^[11–13]^ and sarcopenia.^[20–22]^ The phase angle is commonly defined as the angle which resistance (intracellular and extracellular) and reactance (cell membrane-specific resistance) form. Muscles includes higher the water contents resulting in lower resistance.^[11]^ Our study demonstrated that PA irrespective of the presence of sarcopenia affected mortality in patients undergoing MHD. This suggests that other components except for muscle is important to form PA, and indirectly demonstrates that PA measured by BIA might be much better in assessing clinical prognosis in these patients.

Although the PA has shown prognostic potential in several studies, its relevance as a simple predictor of morbidity in MHD patients has rarely been reported.^[29,30]^ It was shown that reactance (measured by BIA), one of the major components of PA, was a reliable independent predictor of hospitalization in MHD patients.^[29]^ Beberashvili et al demonstrated that the rate of hospitalization due to heart failure significantly decreased (by 21%) with every 1° increase in the PA. However, the prognostic value of low PA could be reduced depending on the SMI. They suggested that the PA reflects both nutritional status and other important prognostic factors.^[30]^ Unlike for mortality, no association was found between PA and MACE in our study, which did not include patients hospitalized due to heart failure. This may have been due to the retrospective nature of the study. Medical records pertaining to MACE may have been deleted or overlooked, whereas deaths are clearly documented, for example by death certificates. The relatively short follow-up period may also have played a role in the lack of an association between PA and MACE. Overall, however, this study is significant in that it is the first to evaluate whether PA is a major independent predictor of MACE.

Some limitations of our study should be considered. First, it used an observational design; the lack of manipulation of variables did not allow for cause-and-effect relationships between outcomes and risk factors to be established. Second, the data on cardiovascular death, which is an important component of MACE, might not have been accurate, because the cause of death was only documented in medical records and death certificates. Other components of MACE may have been similarly underestimated. For example, several events that occurred in regional HD units may have been omitted. Third, two components of sarcopenia, strength and physical performance, were not assessed. Finally, we only analyzed values on patient enrollment; changes over follow-up were not evaluated. A prospective study manipulating several variables will be needed to overcome these limitations.

In conclusion, the PA value, as derived by BIA, was well correlated with several nutritional markers. PA measured by BIA was a good marker of survival in MHD patients, regardless of sarcopenia. The BIA-derived PA could help clinicians to identify and monitor at-risk MHD patients and provide them with active medical and nutritional support. Intervention studies are needed to confirm if the improvement in PA is associated with better clinical outcome.

## Acknowledgments

The authors would like to thank all the colleagues who participate in this project.

## Author contributions

**Conceptualization:** Eunjin Bae and Tae Won Lee

**Data curation:** Wooram Bae, Seongmin Kim, and Jungyoon Choi

**Formal analysis:** Ha Nee Jang and Se-Ho Chang

**Investigation:** Se-Ho Chang and Dong Jun Park.

**Methodology:** Eunjin Bae and Tae Won Lee

**Supervision:** Se-Ho Chang and Dong Jun Park.

**Validation:** Tae Won Lee, Eunjin Bae, and Ha Nee Jang

**Writing – original draft:** Eunjin Bae, Ha Nee Jang, and Tae Won Lee

**Writing – review & editing:** Eunjin Bae and Dong Jun Park.
